# Serum tumor markers in pediatric osteosarcoma: a summary review

**DOI:** 10.1186/2045-3329-2-9

**Published:** 2012-03-23

**Authors:** Yulia A Savitskaya, Genaro Rico-Martínez, Luis Miguel Linares-González, Ernesto Andrés Delgado-Cedillo, René Téllez-Gastelum, Alfonso Benito Alfaro-Rodríguez, Antonio Redón-Tavera, José Clemente Ibarra-Ponce de León

**Affiliations:** 1 Tissue Engineering, Cell Therapy and Regenerative Medicine Unit, National Institute of Rehabilitation, Calzada México Xochimilco 289, Colonia Arenal de Guadalupe, Delegación Tlalpan, México, D.F., México, Código Postal 14389; 2 Department of Bone Tumors, National Institute of Rehabilitation, Calzada México Xochimilco 289, Colonia Arenal de Guadalupe, Delegación Tlalpan, México, D.F., México, Código Postal 14389; 3 Laboratory of Clinical Pathology, National Institute of Rehabilitation, Calzada México Xochimilco 289, Colonia Arenal de Guadalupe, Delegación Tlalpan, México, D.F., México, Código Postal 14389; 4 Department of Chromatography, National Institute of Rehabilitation, Calzada México Xochimilco 289, Colonia Arenal de Guadalupe, Delegación Tlalpan, México, D.F., México, Código Postal 14389; 5 Department of Pediatric Orthopedics, National Institute of Rehabilitation, Calzada México Xochimilco 289, Colonia Arenal de Guadalupe, Delegación Tlalpan, México, D.F., México, Código Postal 14389; 6 Department of Sports Medicine and Arthroscopy, National Institute of Rehabilitation, Calzada México Xochimilco 289, Colonia Arenal de Guadalupe, Delegación Tlalpan, México, D.F., México, Código Postal 14389

**Keywords:** pediatric osteosarcoma, serum tumor markers, natural immunity, thyroid hormone homeostasis, bone tumorigenesis

## Abstract

Osteosarcoma is the most common primary high-grade bone tumor in both adolescents and children. Early tumor detection is key to ensuring effective treatment. Serum marker discovery and validation for pediatric osteosarcoma has accelerated in recent years, coincident with an evolving understanding of molecules and their complex interactions, and the compelling need for improved pediatric osteosarcoma outcome measures in clinical trials. This review gives a short overview of serological markers for pediatric osteosarcoma, and highlights advances in pediatric osteosarcoma-related marker research within the past year. Studies in the past year involving serum markers in patients with pediatric osteosarcoma can be assigned to one of four categories, i.e., new approaches and new markers, exploratory studies in specialized disease subsets, large cross-sectional validation studies, and longitudinal studies, with and without an intervention.

Most of the studies have examined the association of a serum marker with some aspect of the natural history of pediatric osteosarcoma. As illustrated by the many studies reviewed, several serum markers are emerging that show a credible association with disease modification. The expanding pool of informative osteosarcoma-related markers is expected to impact development of therapeutics for pediatric osteosarcoma positively and, it is hoped, ultimately clinical care. Combinations of serum markers of natural immunity, thyroid hormone homeostasis, and bone tumorigenesis may be undertaken together in patients with pediatric osteosarcoma. These serum markers in combination may do better. The potential effect of an intrinsic dynamic balance of tumor angiogenesis residing within a single hormone (tri-iodothyronine) is an attractive concept for regulation of vascularization in pediatric osteosarcoma.

## Introduction

Osteosarcoma is the most prevalent malignant bone tumor [1]. Accounting for 30%-80% of the primary skeletal sarcomas, it is the most common of all bone malignancies [2]. The population affected is predominantly children, teenagers, and young adults aged 10-30 years [3]. Males are more affected than females. Osteosarcomas predominantly target the long cylindrical bones, including the knee joint (approximately half of the observations) and the humerus [4]. Among the most affected are the femur, tibia, and humerus. The tumor is less frequently localized in the shoulder blade, pelvic, and skull bones [5]. Typically, osteogenetic sarcomas metastasize early into the lungs, and metastases to lymph nodes are found in isolated cases [6,7].

Today's leading researchers, pharmacy and biotechnology decision-makers, technology companies, and clinicians are focusing on the use of serum tumor markers in the field of pediatric osteosarcoma research. The search for serum tumor markers for early detection and diagnosis of pediatric osteosarcoma has been a daunting task which has been met with little success. Much of the research effort in the past has been centered on discovery and characterization of single markers [8-10]. A marker is applicable as a fluid analyte when at least two requirements are met, i.e., the potential marker must circulate in the serum and a quantitative high-throughput assay must be available to detect the marker.

As a rule, the types of cancer that develop in children are different from the types that are found in adults. Various international osteosarcoma study groups, including the Cooperative German-Austrian-Dutch Osteosarcoma, National Osteosarcoma Etiology, North American Developments in Osteosarcoma, European American Osteosarcoma, Scandinavian Sarcoma, and North American Children's Oncology groups, have agreed on trying to conduct a collaborative tumor marker study [11-16]. The power of such collaboration lies in the ability to conduct large research projects for studying pediatric osteosarcoma.

This paper gives an overview of established and experimental serological markers in the diagnosis of pediatric osteosarcoma. We have focused exclusively on osteosarcoma in the younger age group. This review summarizes mainly the prognostic values of classical and novel markers for pediatric osteosarcoma.

### Serum tumor marker discovery offers hope of new pediatric osteosarcoma test

A wide variety of serological markers have been associated with pediatric osteosarcoma (Table 1). These may be broadly divided into several groups. Markers are most commonly grouped by chemical structure or by the biological function they have in the organism [17-19]. Chemically, markers can be divided into glycoproteins, polypeptides, carbohydrate determinants of glycoproteins, glycolipids, proteins, polyamines, and immunoglobulins [20-23]. In terms of biological function, markers can be divided into oncofetal antigens, enzymes, hormones, receptors, and compounds with an as yet unclear function [24-26]. Tumor markers involved in angiogenesis, cell adhesion, apoptosis, and the cell cycle have been shown recently to play an important role in osteosarcoma growth, differentiation, and metastasis [27-30]. Over the coming years, the new markers may be able not only to prognosticate pediatric osteosarcoma patients at baseline but also to serve as therapeutic targets and thereby further improve survival rates [31-33]. No osteosarcoma-specific marker, more particularly pediatric osteosarcoma-specific marker, has been found thus far, so where lies the future of pediatric osteosarcoma biomarker research?

**Table 1:** List of candidate serum markers for pediatric osteosarcoma and their possible clinical utility.

Serum marker	Observation for pediatric osteosarcoma	Assessed clinical utility for pediatric osteosarcoma	References
Free polyamines	POS development is accompanied by disorders of polyamine metabolism spurring their intensive release from cells into biological fluids	Informative indicator of a malignant process in POS	Ladanyi et al [35]

IGF-1 and IGFBP-3	IGF-1/IGFBP-3 levels correlate with the presence of metastatic disease, histologic response, event-free survival	Promising predictive factor of development or clinical characteristics of POS	Rodriguez-Galindo et al [36]

anti-ki57 antibody	Increased levels anti-ki57 antibody associated with extent of biological activity of tumor and clinical course of POS	Prognostic factor for POS progression	Petrosyan et al [37]

TNF-β and sTNF-R	In high-grade POS, high levels of TNF-β correlated with bad response to neoadjuvant chemotherapy	Marker for monitoring of response to neoadjuvant chemotherapy in POS	Holzer et al [38]

ANG	Expression of ANG correlates with an increase in local density of blood vessels in tumor tissue, with development of pulmonary metastasis and poor prognosis	Diagnostic and prognostic factor of primary POS	Kushlinskii et al [52]

Bone formation/resorption	Decreased production of PICP, OC, ICTP associated with bone metabolism in POS	Risk factor for pathologic bone fractures in POS	Gajewska et al [40]

T3	Increased levels T3 associated with poor/good disease-free survival	Marker of good and poor POS prognosis	Sidorenko et al [42]

CD44	No significant difference was observed between serum CD44 levels of children with sarcoma and healthy children	Serum CD44 levels were not found to be of value in diagnosis or prognosis for POS	Kebudi et al [43]

VEGF	Increased VEGF levels correlates with tumor stage and disease-free survival	Prognostic factor in POS	Koznetsova et al [85]

SAA	Increased SAA levels associated with type of tumor and high-risk POS development	Differentiates malignant bone cancer from benign bone tumors and detects POS in high-risk children	Krizkova et al [44]

BALP	Increased BALP levels associated with development of POS	Marker for late detecting, monitoring, and assessment of the efficacy of therapy in POS	Ambroszkiewicz et al [17]

CXC chemokines	Increased CXCL4, CXCL6, and CXCL12 levels associated with poor disease-free survival	Prognostic factor for POS outcomes	Li et al [50]

IL-2, IL-4, IL-8, IFN-γ, TNF-α	Analysis of cytokine concentration showed large statistically significant differences between POS and control group for IL-4 and IL-8	Markers for individual reaction of organism to the development of POS	Markiewicz et al [24]

### Free polyamines

One known method of diagnosing cancer is to use free polyamines as biochemical tumor markers in children's osteosarcoma [34]. Tumor development is accompanied by disorders of polyamine metabolism, spurring their intensive release from cells into biological fluids (blood and urine). Based on this, the level of free polyamines is measured in blood sera and blood-formed elements. Their levels in children with osteosarcoma were found to exceed reference values in 92.4% of cases for formed elements and 63% for plasma [35]. Following tumor surgery, 58% of children showed a decrease in polyamine count. The data suggest that measurement of free polyamines in blood-formed elements on a background of cancer can be used before treatment as an informative indicator of a malignant process. The fact that removal of tumor locality fails to normalize polyamine levels in up to half of cases points to the shortcomings of this method when the polyamine level has to be used in treated patients to test for recurrence.

### Insulin-like growth factor-1 and insulin-like growth factor binding protein-3

Preclinical work has suggested a role of insulin-like growth factor-1 (IGF-1) in the proliferation of osteosarcoma cells in vivo [36]. This research group has addressed the relationship between serum levels of IGF-1, its binding protein (IGFBP-3), and the clinical behavior and outcome of osteosarcoma in children. In a retrospective study of 37 patients, it was found that circulating levels of IGF-1 and IGFBP-3 were not predictive of the development or clinical characteristics of pediatric osteosarcoma. However, further studies in a larger patient population should be performed in order to investigate this relationship further.

### Monoclonal antibody of circulating tumor-associated antigen ki67

A prognostication method in children's osteosarcoma using ki67 monoclonal antibodies is known. Using these antibodies, Petrosyan et al [37] found that the mean binding index in bone sarcomas is 9.7% and markedly exceeds the same parameter in benign bone neoplasms. The expression of this antigen has been shown to correlate with the extent of biological tumor activity and the clinical course of the disease. However, studies into the possibility of using this indicator as a prognostic test preceding recurrences have not been conducted, rendering its application in this respect impossible at present.

### TNF-β and soluble TNF receptor

Kotz et al [38] determined serum levels of tumor necrosis factor beta (TNF-β) and soluble TNF receptor in pediatric patients with highly malignant primary bone tumors. Both TNF-β and soluble TNF receptor levels were lower in serum from pediatric osteosarcoma patients as compared with those with Ewing's sarcoma. In patients with high-grade osteosarcoma, but not Ewing's sarcoma, high levels of TNF-β correlated with a bad response to neoadjuvant chemotherapy. In patients with high-grade osteosarcoma, TNF-β levels seem to be of predictive value, and both TNF-β and soluble TNF receptor levels seem to be of diagnostic value for differentiation between high-grade osteosarcoma and Ewing's sarcoma in children.

### Markers of bone formation and resorption

In adolescent patients with bone sarcoma, bone mass acquisition is potentially compromised at a time when it should be at a maximum [39]. To evaluate this problem, the authors measured bone mineral density and serum markers of bone formation and resorption in a series of pediatric patients with bone tumors [40]. Ruza et al [41] analyzed 85 samples from 59 patients with osteosarcoma and 54 samples from 36 patients with Ewing's sarcoma. Serum markers of bone formation (procollagen type I C-terminal propeptide and osteocalcin) and resorption (carboxyterminal telopeptide) were lower than reference values throughout. Significant alterations in other markers were also observed. Up to one third of patients with osteosarcoma and Ewing's sarcoma in clinical remission had some degree of bone mineral density deficit. The corresponding increased risk in pathologic bone fractures heralds a reduction in future quality of life.

### Tri-iodothyronine

The functional condition of different components of the endocrine system undergoes certain changes during the malignant process. In several situations, it was determined that an impaired hormonal balance can have a central role in the pathogenesis of tumoral transformation in the tissues. This suggests that the search for dynamic deviations in levels of biologically active compounds developing during the premanifestation stage needs to be conducted for hormonal indicators, in particular indicators of thyroid homeostasis, since it is the thyroid hormones that, unlike other hormones, have the widest range of biological activity, including regulating the main stages of all metabolic processes in the organism, affecting proliferation speed and nervous and immune system function, the status of which, in turn, largely determines resistance of the organism to malignancy [42]. A method of predicting process generation in children and adolescents with osteosarcoma useful for comprehensive tumor treatment and biochemical study. One distinctive feature is that after comprehensive treatment resulting in a complete clinical and laboratory remission, the patient's blood is tested for the free fraction of tri-iodothyronine thyroid hormone. A level of 4.0-5.2 pmol/mL predicts remission of 6-24 months, while a level of 5.8-7.4 pmol/mL points to generation of the malignant process in 1-3 months. The assessment makes it possible to use free T3 in the blood after treatment as an additional laboratory indicator to assess the future course of the disease and to initiate appropriate treatment in a timely fashion.

### CD44

Kebudi et al [43] compared serum CD44 levels in children having sarcomas with those in healthy children. There was no statistically significant difference between serum CD44 levels in children with sarcomas and those in healthy children. There was no significant difference between serum CD44 levels according to stage or outcome. In this study, serum CD44 levels were not found to be of diagnostic or prognostic value for osteosarcoma in children.

### Amyloid protein A

Using proteomic profiles, the authors constructed a multivariate three-nearest neighbor classifier to distinguish pediatric osteosarcoma from osteochondroma patients with a sensitivity of 97% and a specificity of 80% based on external leave-one-out cross-validation [44]. One of the proteins in the proteomic signature was identified as serum amyloid protein A. Classification based on this plasma proteomic signature may be useful to differentiate malignant bone cancer from benign bone tumors and for early detection of osteosarcoma in high-risk children.

### Bone alkaline phosphatase

Osteosarcoma usually develops in young children [45]. While the incidence of osteosarcoma increases steadily with age, a relatively dramatic increase in adolescence corresponds with the growth spurt [46]. This is good reason to suggest a link between the blastomatic process and the bone growth spurt, as well as sports and other physical activities. Biochemical markers of bone turnover reflecting the intensity of the bone remodeling process in the skeleton are important for fast and noninvasive assessment of bone formation and resorption processes [47]. They can be used in terms of both physiological and pathological states. An elevated level of serum bone alkaline phosphatase, which is found in more than 40% of osteosarcoma patients, is also a valuable diagnostic parameter [48]. However, due to the difficulties in general standardization, this parameter may be difficult to interpret in younger patients. Ambroszkiewicz et al showed that bone turnover markers, especially bone alkaline phosphatase, may be useful in the monitoring and assessment of treatment efficacy in children's osteosarcoma [49].

### CXC chemokines

Li et al [50] used an antibody microarray to identify chemokines that were elevated in serum samples of pediatric osteosarcoma patients. The results demonstrated that CXCL4 and CXCL6 are frequently expressed in osteosarcoma, and that plasma levels of these two chemokines are associated with patient outcomes. Further study of these circulating chemokines may provide a promising approach for prognostication of pediatric osteosarcoma.

### Cytokines

Causes of osteosarcoma are still unknown and the reaction of the immune system to its development is very individual. Particular emphasis must be placed on the role of cytokines in immunoregulatory and coordinating functions and tumor cell disruption. Knowledge about cytokine concentrations in serum, with regard to mechanisms of oncogenesis, may have prognostic significance for the further course of osteosarcoma in children. In a study that evaluated interleukin (IL)-2, IL-4, IL-8, interferon (IFN)-γ, and TNF-α concentrations at the time of diagnosis in children with osteosarcoma, the following concentrations of peripheral blood cytokines were observed: IL-2 10.7 pg/mL, IFN-γ 1.3 pg/mL, TNF-α 28.3 pg/mL, IL-4 2.0 pg/mL, and IL-8 13.5 pg/mL [51]. Results of studies obtained at diagnosis did not give a specific answer about the prognosis and further course of osteosarcoma in patients according to age. Big differences in cytokine concentrations in children and youth with osteosarcoma might be associated with individual biological variations and individual reactions to the development of neoplastic disease. Further studies in this direction are needed before the start of cytostatic therapy and therapeutic monitoring of cytostatic therapy.

### Angiogenin

The relevance of neoangiogenesis (formation of new blood vessels) in malignant tumors is not in doubt, because tumors cannot develop without forming an extensive capillary network to ensure the performance of their vital functions [52,53]. A critical step in the development of osteosarcoma is hematogenous metastasis [54]. The average dissemination time does not usually exceed 8-12 months. The results of many study confirm the suggestion that angiogenin plays a key role in hematogenous metastasis [55]. The development of angiogenin-dependent angiogenesis and the growth of small blood vessels increases the chances of tumor cells entering the circulation, because the newly formed small capillaries with a fragmented basal membrane are much easier for the tumor cells to pass through than larger vessels [56]. In addition, a large number of small vessels have a larger surface through which tumor cells metastasize quickly. Correlation between the angiogenin level in the blood and the presence of metastasis, as well as the timing of metastasis, appears to have prognostic significance in osteosarcoma patients [57]. Thus, the expression of angiogenin in primary osteosarcoma correlates with an increase in the local density of blood vessels in tumor tissue, with the development of metastatic pulmonary disease and a poor prognosis [58]. It may be possible to use angiogenin as a marker for predicting metastasis in pediatric osteosarcoma patients. This is important due to the fact that diagnostic errors, as experience shows, are fairly common.

A study by Kreuter et al assessed osteosarcoma tumorigenesis in pediatric patients treated on the basis of microvessel density and vascular endothelial growth factor expression [59]. No differences in the extent of angiogenesis were seen in relation to treatment outcome or presence of metastasis. However, studies like this are scarce and the results are not always accurate.

### Biomarker-IgM immune complexes

Natural antibodies are hallmark components of anticancer activity [60,61]. Natural antibodies have multiple roles in the immune system, [62] and appear to maintain B cell homeostasis, clear apoptotic cells, and protect against autoimmunity. All tumor-specific antibodies belong almost exclusively to the IgM class [63]. Human serum contains natural antibodies, which are present prior to the development of cancer [64]. During the first phase of this process, i.e., the primary immune response, IgM is the specific antibody produced [65].

In contrast with the detection of serum tumor angiogenesis antigens, the detection of natural serum antibodies to tumor angiogenesis antigens may provide reliable serum markers for early diagnosis and prognosis in pediatric osteosarcoma [66,67]. Tumor-associated natural IgM antibodies circulate in the blood much earlier than serum antigens [68,69]. Natural IgM antibodies to tumor antigen have been reported in patients with early-stage cancer, and a panel of serum antibodies can detect cancer 5 years prior to radiographic detection [70,71]. Biomarker-IgM immune complexes are a novel class of serum tumor markers with greater diagnostic potential than the corresponding free markers.

The advantage of this approach is that it should be possible to detect the full range of heterogenous osteosarcomas by increasing the number of angiogenin-IgM assays or altering the antigens used in the natural IgM antibodies panel. Circulating immune complexes formed by tumor antigens and IgM represent a novel class of serum tumor markers with diagnostic value for early detection of osteosarcoma. Measuring natural antibodies to angiogenesis bioregulators could become an additional method for early diagnosis of pediatric osteosarcoma. Analysis of the work done to date will allow the physician to assess the health status of a child objectively, to obtain data on possible risk groups based on selected criteria and initiate prevention efforts in a timely manner. Studies in these areas have become the basis of a new research direction for comprehensive study of immune status in children [72]. Maintenance of angiogenin-IgM may also be an important aspect of osteosarcoma tumorigenesis and could be linked to prognosis. It is thus reasonable to increase the range of classical markers using in practice by adding such novel prognostic markers as angiogenin-IgM.

### Potential use of serum tumor markers for early detection, diagnosis, and prevention

We do not know what causes children to develop bone tumors. Only about two in every 10 children who develop malignant bone tumors have a family history of the condition [73]. Children aged 6-12 years are the age group most likely to develop benign bone tumors, although the tumors sometimes show up in children as young as two years of age [74]. Osteosarcoma usually begins when children are young teenagers [75]. This is a time when their bones are growing very rapidly and they are often taking part in sports and other physical activities [76]. The most common age at which children are diagnosed with these cancers is 14 years [77].

Progress in the fields of biochemistry, molecular biology, and biotechnology has allowed researchers and clinicians to use biologically significant indicators to assist in predicting early osteosarcoma in children and in choosing appropriate therapy when the disease is advanced. The future of pediatric osteosarcoma markers must involve a more molecular approach to the measurement of relevant clinical prognostic factors and the development of treatments based on the molecular profile of tumors.

The specificity of an individual tumor marker is quite low, but increases when several markers are used in combination [78,79]. The accuracy of studies of tumor markers is contingent on their sensitivity and specificity [80-83]. Markers can be major, minor, and additional [84]. A major marker is one with high sensitivity and specificity for a certain type of tumor [85]. Minor markers are typically measured in parallel with measurement of the major marker [86]. Minor markers have lower tumor sensitivity and specificity in comparison with the major marker. When used in conjunction with the major marker, a minor marker increases the probability of detecting a tumor. Additional markers are generally more sensitive to the disease than minor markers, but can be specific to a particular organ. In addition, the increase in marker levels is associated with tumor recurrence [87].

Because osteosarcoma is heterogeneous, we suggest measuring all three types of markers at the beginning of treatment (marker of natural immunity as major, marker of thyroid hormone status as minor, and marker of bone tumor angiogenesis as additional). The triple test is an investigation to classify a pediatric patient as either high-risk or low-risk for osteosarcoma. An understanding of the balance between, bone tumor angiogenesis, and thyroid hormone status may assist in diagnostics and therapeutics for children with osteosarcoma. The potential effect of an intrinsic dynamic balance of tumor angiogenesis residing in a single hormone is an attractive concept for regulation of vascularization in osteosarcoma. A positive test in children indicates a high risk of developing osteosarcoma.

## Discussion

The following conclusions (Table 1) can be drawn after analyzing the data on the significance of serum tumor markers for diagnosing pediatric osteosarcoma (early diagnosis, differential diagnosis, early detection of recurrence and metastasis), monitoring (assessment of extent of tumor, choice of adequate therapy, evaluating effectiveness of treatment) and prognosis [88-92]:

● So far no specific diagnostic preparation has been developed capable of detecting a malignant tumor of a certain histological type (pediatric osteosarcoma) and locating it at the earliest possible stages of formation.

● No universal and specific serum tumor markers have been found for early diagnosis of pediatric osteosarcoma and primary screening in risk groups.

● Several markers can be used successfully for the diagnosis of osteosarcoma in children.

● Most of the characterized markers are used for monitoring, while some are employed for the prediction of pediatric osteosarcoma.

● Although initial expectations concerning sensitivity and specificity of individual serum markers have not been fulfilled, a rational approach to these tests and balanced interpretation of the results make them clinical significant.

● The number of serum markers for diagnosing osteosarcoma is growing, which poses the question of differentiating between them, performing complex multivariate analysis of diagnostic tests pathognomonic for osteosarcoma, the use of discriminant variables, which will optimize the examination plans for individual patients, monitoring, and giving a prognosis.

We must acknowledge that, despite the presence of a broad range of tumor markers for the diagnosis and monitoring of pediatric osteosarcoma, so far no unequivocal system has been developed for introducing these markers into clinical practice. Based on analysis of the current state of the problem of using tumor markers for primary diagnosis and monitoring in children's osteosarcoma, schemes have been developed for the most effective and appropriate serum markers. These schemes include nonspecific tumor-induced and tumor-produced markers with high specificity and sensitivity that have been found suitable for topical diagnosis. Introduction of biochemical tests for diagnosis and monitoring should be decided at the international level with regard to equipment in laboratories. Nonspecialized laboratories tasked with surveying the patient for diagnosis, in particular the identification of pathology, can be advised to use simple, affordable systems for nonspecific markers [93-95].

The clinical significance of individual serum osteosarcoma markers has been widely debated. Despite the fact that the relevance of these markers is being checked constantly, some of them are used in clinical practice. The importance of individual tumor markers need not be overstated, since they are only an additional diagnostic tool with limited applicability and diagnostic accuracy. Improving their diagnostic accuracy requires an individual therapeutic approach to each patient. Formulation of an individual approach is largely determined by the choice of tactics based on knowledge of the major factors influencing the course of the disease. Numerous international studies conducted using univariate and multivariate analysis revealed a number of prognostic factors in osteosarcoma.

Serum tumor markers seemed to be ideal for early diagnosis of cancer. However, the lack of sensitivity and specificity has been a major problem in the use of most serum tumor markers for diagnosis of pediatric osteosarcoma. In the vast majority of research studies over the past two decades, only combination of major (natural immunity), minor (thyroid hormone status), and additional (bone tumor angiogenesis) markers has been applied as a "gold standard" for monitoring and diagnosis of pediatric osteosarcoma patients. Recent advances in knowledge concerning the molecular biology of pediatric osteosarcoma will hopefully result in serum tumor markers.

## Abbreviations

ANG: angiogenin; BALP: bone alkaline phosphatase; IL: interleukin; TNF: tumor necrosis factor; POS: pediatric osteosarcoma; IFN: interferon; SAA: serum amyloid protein A; IGF-1: insulin-like growth factor-1; IGFBP-3: insulin-like growth factor-1 binding protein; VEGF: vascular endothelial growth factor; sTNF-R: soluble TNF receptor; PICP: procollagen type I C-terminal peptide; OC: osteocalcin; ICTP: pyridinoline cross-linked carboxyterminal telopeptide of type I collagen.

## Competing interests

The authors declare that they have no competing interests.

## Authors' contributions

YAS conceived of the manuscript aims, reviewed the literature and wrote the manuscript. GRM co-designed the manuscript. LMLG corrected the writing. EADC reviewed the literature. RTG checked the serum biomarkers content. ABAR edited the manuscript. ART assisted in revision of the manuscript. JCIPL directed the manuscript. All authors read and approved the final manuscript.
